# Prevalence of eye diseases among prison inmates in Sub-Saharan Africa: a systematic review and meta-analysis protocol

**DOI:** 10.3389/fmed.2026.1882529

**Published:** 2026-07-16

**Authors:** Josephine Ampong, Emmanuel Tettey Doku, Werner Eisenbarth, Eric Tettey Ashong, Kwesi Sewe, Bismark Beesi Coffie, Kelvin Oppong Kyeremateng, Isaiah Osei Duah Junior, Albert Kwadjo Amoah Andoh, David Ben Kumah, Hornametor Yao Afake, Kwadwo Owusu Akuffo

**Affiliations:** 1 Department of Optometry and Visual Science, College of Science, Kwame Nkrumah University of Science and Technology, Kumasi, Ghana; 2 Department of Applied Science and Mechatronics, HM Hochschule München University of Applied Sciences, Munich, Germany; 3 University of Ghana Library System, University of Ghana, Accra, Ghana; 4 Department of Psychology, John R. and Kathy R. Hairston College of Health and Human Sciences, North Carolina Agricultural and Technical State University, Greensboro, NC, United States; 5 National Eye Care Unit, Ghana Health Service, Accra, Ghana

**Keywords:** blindness, eye diseases, meta-analysis, ocular morbidity, prison inmates, Sub-Saharan Africa, systematic review, visual impairment

## Abstract

**Background:**

Eye diseases constitute a major public health challenge globally, with a disproportionate burden borne by populations in low- and middle-income countries (LMICs), including Sub-Saharan Africa (SSA). Prison populations represent a uniquely vulnerable group characterized by restricted access to healthcare, overcrowding, poor nutrition, and high-risk environmental conditions — all of which substantially elevate the risk of eye diseases. Despite individual country-level reports documenting elevated rates of eye disease among incarcerated persons in SSA, no synthesis of the evidence has been conducted. This systematic review and meta-analysis protocol aims to consolidate existing evidence on the prevalence of eye diseases among prison inmates across SSA.

**Methods:**

The protocol has been developed in accordance with the Preferred Reporting Items for Systematic Reviews and Meta-Analyses Protocols (PRISMA-P) guidelines to ensure rigor, transparency, and reproducibility. We will search major databases, including PubMed, Scopus, Web of Science, Embase, CINAHL (via EBSCO), PsycINFO, Google Scholar, and African Journals Online, using controlled vocabularies without date or language restrictions. Risk of bias and study quality will be assessed using standard critical appraisal tools. The completed review will follow the PRISMA reporting checklist and where appropriate, a meta-analysis will be conducted to generate pooled estimates, with subgroup analyses to explore sources of heterogeneity and country-level variations.

**Discussion:**

Given the eye’s role as a non-invasive window into systemic and overall health, findings from this review may inform evidence-based screening strategies and support policy reforms aimed at improving ocular and general health care in prison settings.

**Study protocol registration:**

https://www.crd.york.ac.uk/PROSPERO/view/CRD420261389357, identifier (CRD420261389357).

## Background

According to the Global Burden of Disease Study 2020, an estimated 43.3 million people were blind worldwide, with a further 295 million experiencing moderate-to-severe vision impairment (1). The SSA is disproportionately affected with approximately five million people in the region living with blindness and about 20 million having moderate-to-severe vision impairment (2). Despite significant progress in addressing avoidable blindness globally, prison inmates have remained one of the most underserved and neglected groups in the global eye health landscape (3–5). There are currently over 6 million prisoners in SSA, and these populations experience more complex health needs and carry a heavier burden of both communicable and non-communicable diseases compared with the general population (6, 7). Across SSA, correctional facilities are characterized by severe overcrowding, with many institutions operating at two to five times their designed capacity, creating environments of poorly ventilated spaces with inadequate sanitation (8–10). These physical conditions are compounded by nutritional deficiencies arising from poor-quality prison rations and limited dietary diversity (11–13). Prisoners also face restricted access to healthcare services because of understaffing, inadequate medical infrastructure, and systemic neglect (14–16). In addition, incarceration exposes individuals to physical and psychological stressors, including violence, social isolation, and loss of autonomy (17). These factors strongly influence the occurrence, burden and pattern of ocular disease in the prison populations.

There has been reports of high prevalence rates of infectious diseases such as HIV, tuberculosis, and hepatitis, alongside chronic non-communicable diseases including hypertension and diabetes mellitus among prisoners in the region (9, 12, 18, 19). These systemic health burdens have direct implications on ocular health. Conditions such as diabetes, HIV-related opportunistic infections, vitamin A deficiency, hypertension, and tuberculosis are established risk factors for a spectrum of eye diseases, ranging from infectious conjunctivitis and xerophthalmia to diabetic retinopathy and vision-threatening complications (8, 11). Despite this clear association between the prison environment, systemic disease burden, and eye diseases, robust epidemiological data on the prevalence, patterns, and determinants of eye diseases among incarcerated populations in SSA remain scarce (14, 15, 20).

The existing body of evidence on the epidemiology of eye diseases in SSA have focused exclusively on general community populations, providing valuable insights into the burden of ocular conditions among free-living individuals (21, 22). However, these population-based estimates may not be directly applicable to incarcerated populations because health outcomes within prisons are shaped by an interplay of biological, psychological, social, and environmental factors. Biological factors include pre-existing health vulnerabilities, malnutrition, and infectious disease burden; psychological factors include chronic stress, mental health disorders, and limited autonomy, while social and environmental factors include overcrowding, restricted healthcare access, institutional neglect, and poor lighting conditions within correctional facilities (13, 16, 17). Notably, prison populations also tend to include a relatively older demographic compared with the general community, and advancing age is itself a well-established risk factor for several eye diseases (23), further compounding the expected burden of ocular morbidity in this group. Furthermore, the social determinants of health within correctional facilities including institutional food policies, healthcare delivery models, and environmental conditions, differ fundamentally from those in community settings, necessitating context-specific estimates to guide resource allocation and intervention design (12, 13).

Given the substantial environmental and health-system differences between free-living and incarcerated populations, we conjecture that the prevalence of eye diseases among prison inmates in SSA will be higher than in the general population, with a distinct etiological profile shaped by the conditions of incarceration. A systematic review and meta-analysis is therefore essential to synthesize available evidence on eye disease burden among incarcerated populations in SSA. This review aims to determine the reported prevalence of eye diseases among prison populations in SSA, characterize the ocular conditions reported among prisoners, examine associated risk factors, and summarize the diagnostic and screening methods used in prison eye health studies. By systematically aggregating data from individual facility-level studies, this review will provide pooled prevalence estimates, explore variations across geographic regions and correctional settings, and identify gaps in the provision of eye care services. Furthermore, assessing the methodological quality of existing evidence will help identify limitations in current research and inform future investigations. Overall, the findings from this review will help inform the development of tailored screening protocols, treatment guidelines, and health system strengthening initiatives that address the distinct needs of this vulnerable and often neglected population.

## Methods

The protocol was prepared in accordance with the guidelines of the PRISMA-P (Preferred Reporting Items for Systematic Reviews and Meta-Analyses Protocol) (24), see Supplementary File 1. The findings of the review will be reported following the PRISMA checklist (Preferred Reporting Items for Systematic Reviews and Meta-Analyses) (25). This protocol has also been indexed in the International Prospective Register of Systematic Reviews (PROSPERO ID: CRD420261389357).

### Eligibility Criteria

#### Inclusion criteria

##### Population

The review will include studies conducted among adult and juvenile incarcerated populations. Juvenile individuals will be defined according to the legal and contextual definition applied in the country where the study was conducted. Where studies provide age-specific information, juvenile populations will include individuals below 18 years of age, while adult populations will include individuals aged 18 years and above. No maximum age limit will be applied. Both male and female inmates will be included.

##### Condition of interest

Studies must report data on the prevalence of at least one of the following: any eye disease; specific conditions including refractive errors (myopia, hyperopia, astigmatism, presbyopia), cataract, glaucoma, conjunctivitis (allergic, infective), trachoma, pterygium, corneal opacities, diabetic retinopathy, hypertensive retinopathy, optic neuropathy, and/or visual impairment and blindness, as defined by the World Health Organization (presenting or best-corrected visual acuity criteria).

##### Operational definitions

The following operational definitions will be applied uniformly across all included studies:

- Ocular morbidity: any clinically identifiable or self-reported condition affecting the eye or visual system, documented in the included study.
- Visual impairment: presenting visual acuity worse than 6/18 in the better eye.
- Blindness: presenting visual acuity worse than 3/60 in the better eye.
- Refractive error: myopia, hyperopia, astigmatism, or presbyopia identified by refraction or documented clinical assessment.
- Infectious ocular disease: conditions including conjunctivitis, trachoma, keratitis, and uveitis with an established infectious etiology.
- Chronic ocular disease: conditions including glaucoma, cataract, diabetic retinopathy, and hypertensive retinopathy with documented chronicity.

Studies relying exclusively on self-reported symptom without any clinical or optometric examination will be flagged during data extraction and analyzed in a separate sensitivity analysis to assess their influence on pooled estimates. Studies using screening-only approaches (e.g., visual acuity screening alone) versus those employing comprehensive ophthalmic examinations will be coded separately in the data extraction form and will be included as a covariate in pre-specified subgroup analyses and meta-regression.

##### Comparators

Where applicable, studies may compare prisoners with eye diseases to those without eye diseases within the prison population. Some studies may also compare different demographic or exposure groups within prison settings, such as age groups, gender categories, or individuals with varying durations of incarceration. Studies that report prevalence of eye diseases without explicit comparator groups will also be considered eligible for inclusion.

##### Outcomes

*Primary outcome:* The prevalence of any eye disease (overall ocular morbidity) among prison inmates.
*Secondary outcomes:* Measures of visual function and ocular health such as visual acuity, prevalence of specific eye conditions, including but not limited to refractive errors, cataract, glaucoma, conjunctivitis, pterygium, visual impairment, and blindness, as well as factors associated with ocular morbidity in this population.

##### Study type

This systematic review will include observational studies that report empirical findings on eye diseases and associated determinants among prison populations in SSA. Eligible study designs will include cross-sectional studies, cohort studies, and case–control studies. These study designs are considered appropriate for estimating prevalence and identifying factors associated with ocular diseases within prison populations.

#### Exclusion criteria

**Figure 1:**
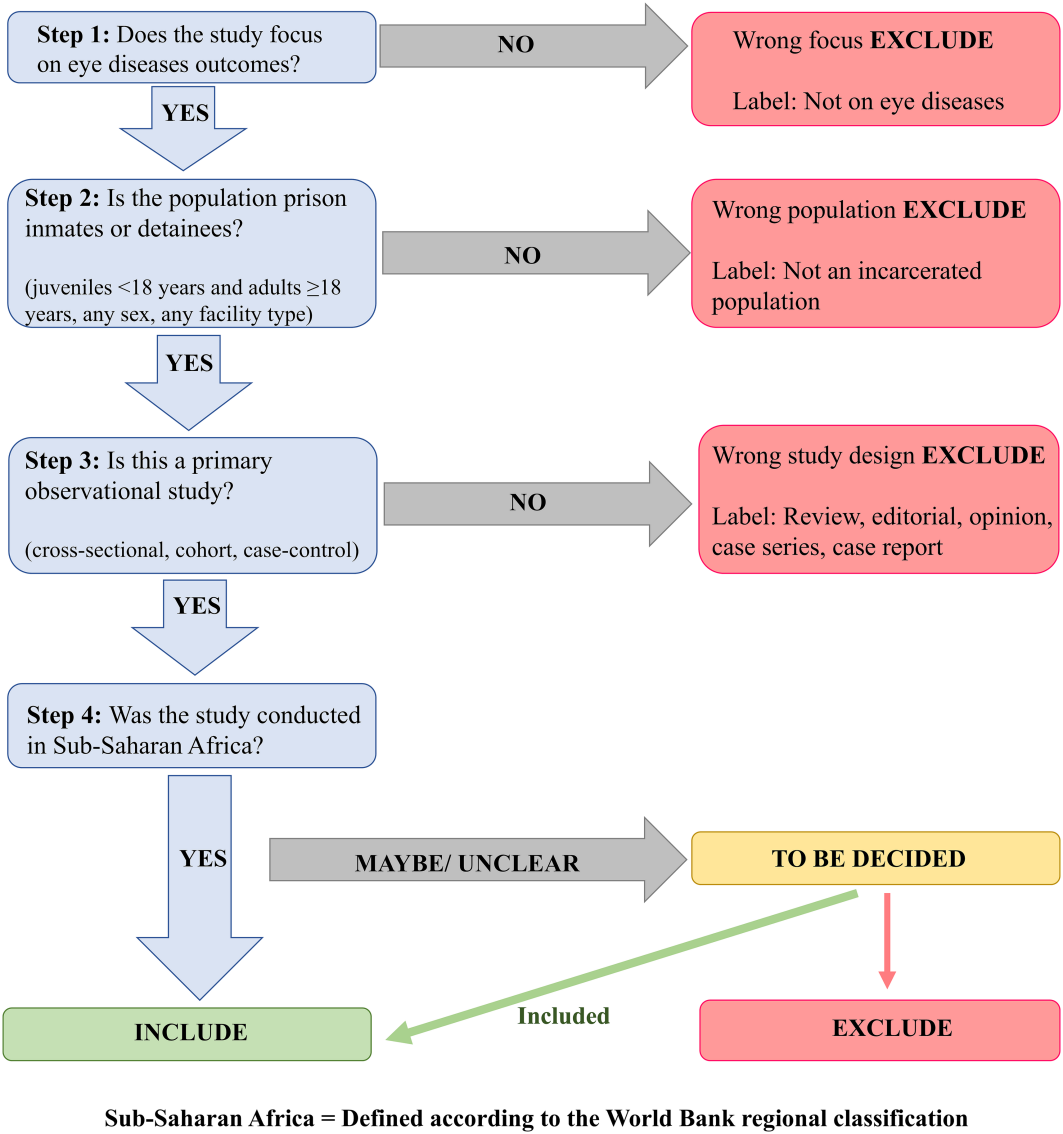
Diagram showing inclusion and exclusion criteria.

Studies will be excluded if they: (1) are conducted outside SSA; (2) do not include incarcerated populations; (3) focus exclusively on correctional staff or non-incarcerated populations; (4) do not report ocular conditions, visual impairment, or eye health outcomes; and (5) are case reports, case series, editorials, commentaries, conference abstracts without sufficient data, or review articles (Figure 1).

### Information sources and search strategy

A comprehensive systematic search will be conducted across the following electronic databases: PubMed, Scopus, Web of Science (Core Collection), African Journals Online (AJOL), Embase, CINAHL (via EBSCO), and PsycINFO. Google Scholar will also be searched and the first 200 results screened for relevant studies not captured by the primary database searches. Backward citation searching of reference lists and forward citation tracking of included studies will be undertaken to identify additional relevant records.

The search strategy has been developed with input from an experienced librarian (K.S) and will use a combination of Medical Subject Headings (MeSH) terms and free-text keywords. The core search terms will be structured around three thematic clusters: (i) prison population; (ii) eye conditions; and (iii) geographic restriction ie. SSA. Boolean operators (AND, OR) will combine clusters, with truncation symbols applied as appropriate to each database (Table 1).

**Table 1:** Search strategy developed in PubMed to be adapted for the other databases.

Search	Search strategy
#1	“prisoners”[MeSH Terms] OR “prison*”[Title/Abstract] OR “prisoner*”[Title/Abstract] OR “inmate*”[Title/Abstract] OR “jail*”[Title/Abstract] OR “gaol*”[Title/Abstract] OR “incarcerat*”[Title/Abstract] OR “detainee*”[Title/Abstract] OR “detain*”[Title/Abstract] OR “detention”[Title/Abstract] OR “custod*”[Title/Abstract] OR “correctional facilit*”[Title/Abstract] OR “correctional institution*”[Title/Abstract] OR “correctional service*”[Title/Abstract] OR “penal institution*”[Title/Abstract] OR “penitentiary”[Title/Abstract] OR “remand”[Title/Abstract] OR “convict*”[Title/Abstract] OR “offender*”[Title/Abstract] OR “prison population*”[Title/Abstract] OR “detection center*”[Title/Abstract] OR “remand home”[Title/Abstract] OR “correctional center*”[Title/Abstract] OR “custodial facility*”[Title/Abstract]
#2	“eye diseases”[MeSH Terms] OR “vision disorders”[MeSH Terms] OR “eye disease*”[Title/Abstract] OR “ocular disease*”[Title/Abstract] OR “ophthalmic disease*”[Title/Abstract] OR “ocular morbid*”[Title/Abstract] OR “eye disorder*”[Title/Abstract] OR “ocular disorder*”[Title/Abstract] OR “visual impair*”[Title/Abstract] OR “vision impair*”[Title/Abstract] OR “low vision”[Title/Abstract] OR “blindness”[Title/Abstract] OR “cataract*”[Title/Abstract] OR “glaucoma”[Title/Abstract] OR “conjunctivitis”[Title/Abstract] OR “trachoma”[Title/Abstract] OR “pterygium”[Title/Abstract] OR “keratitis”[Title/Abstract] OR “corneal opacit*”[Title/Abstract] OR “uveitis”[Title/Abstract] OR “retinopath*”[Title/Abstract] OR “maculopath*”[Title/Abstract] OR “refractive error*”[Title/Abstract] OR “myopi*”[Title/Abstract] OR “hyperopi*”[Title/Abstract] OR “presbyopi*”[Title/Abstract] OR “astigmati*”[Title/Abstract] OR “visual acuity”[Title/Abstract] OR “eye infection*”[Title/Abstract] OR “ocular infection*”[Title/Abstract] OR “ocular trauma”[Title/Abstract]
#3	“Africa”[MeSH Terms] OR “africa south of the sahara”[MeSH Terms] OR “Africa”[Title/Abstract] OR “sub-Saharan Africa”[Title/Abstract] OR “sub-Saharan African”[Title/Abstract] OR “West Africa”[Title/Abstract] OR “East Africa”[Title/Abstract] OR “Central Africa”[Title/Abstract] OR “Southern Africa”[Title/Abstract] OR “Angola”[Title/Abstract] OR “Benin”[Title/Abstract] OR “Botswana”[Title/Abstract] OR “Burkina Faso”[Title/Abstract] OR “Burundi”[Title/Abstract] OR “Cabo Verde”[Title/Abstract] OR “Cape Verde”[Title/Abstract] OR “Cameroon”[Title/Abstract] OR “Central African Republic”[Title/Abstract] OR “Chad”[Title/Abstract] OR “Comoros”[Title/Abstract] OR “Republic of the Congo”[Title/Abstract] OR “Democratic Republic of the Congo”[Title/Abstract] OR “Djibouti”[Title/Abstract] OR “Equatorial Guinea”[Title/Abstract] OR “Eritrea”[Title/Abstract] OR “Eswatini”[Title/Abstract] OR “Swaziland”[Title/Abstract] OR “Ethiopia”[Title/Abstract] OR “Gabon”[Title/Abstract] OR “Gambia”[Title/Abstract] OR “Ghana”[Title/Abstract] OR “Guinea”[Title/Abstract] OR “Guinea-Bissau”[Title/Abstract] OR “Liberia”[Title/Abstract] OR “Kenya”[Title/Abstract] OR “Lesotho”[Title/Abstract] OR “Madagascar”[Title/Abstract] OR “Malawi”[Title/Abstract] OR “Mali”[Title/Abstract] OR “Mauritania”[Title/Abstract] OR “Mauritius”[Title/Abstract] OR “Mozambique”[Title/Abstract] OR “Namibia”[Title/Abstract] OR “Niger”[Title/Abstract] OR “Nigeria”[Title/Abstract] OR “Rwanda”[Title/Abstract] OR “Sao Tome and Principe”[Title/Abstract] OR “Senegal”[Title/Abstract] OR “Seychelles”[Title/Abstract] OR “Sierra Leone”[Title/Abstract] OR “Somalia”[Title/Abstract] OR “South Africa”[Title/Abstract] OR “South Sudan”[Title/Abstract] OR “Tanzania”[Title/Abstract] OR “Togo”[Title/Abstract] OR “Uganda”[Title/Abstract] OR “Zambia”[Title/Abstract] OR “Zimbabwe”[Title/Abstract] OR “Cote d’Ivoire”[Title/Abstract] OR “Ivory Coast”[Title/Abstract]
#4	((#1) AND (#2)) AND (#3)

### Study selection process

**Figure 2:**
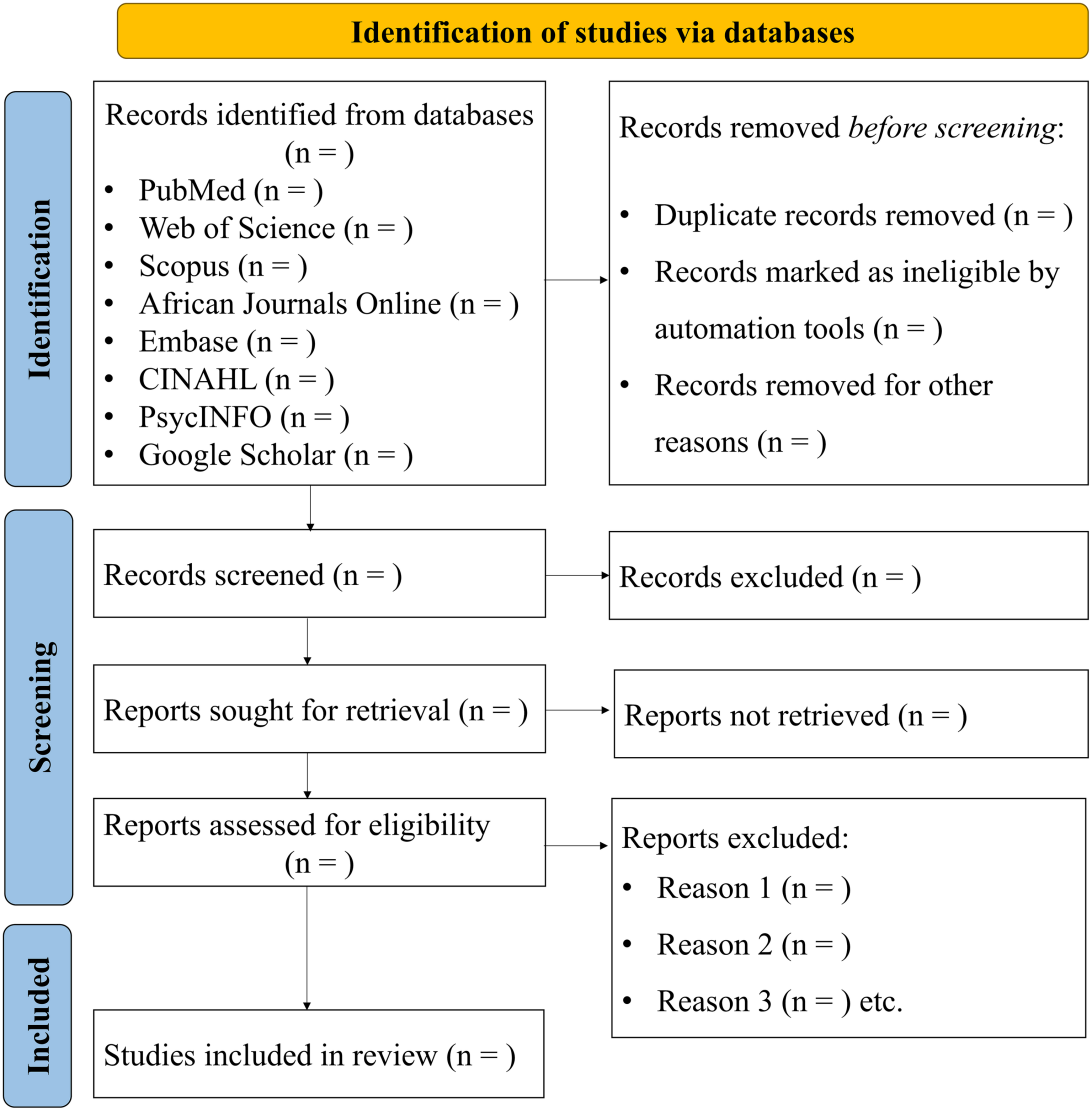
PRISMA flow diagram.

All identified records will be imported into Covidence (26), a web-based systematic review management tool, and duplicates will be removed electronically and manually. Study selection will proceed in two sequential stages against the pre-specified eligibility criteria. In the first stage, two independent reviewers (B.B.C and K.O.K) will screen all retrieved titles and abstracts. Each reviewer will classify each record as “include,” “exclude,” or “maybe.” Disagreements will be resolved by discussion and consensus between the two reviewers; where consensus cannot be reached, a third senior reviewer (J.A) will serve as adjudicator. Records classified as “include” or “maybe” at this stage will be carried forward to full-text screening. In the second stage, all records deemed potentially eligible after title and abstract screening will be retrieved as full texts. Both reviewers will independently assess each full-text article against the eligibility criteria. Reasons for exclusion at this stage will be systematically recorded for each excluded study and will be reported transparently in the PRISMA 2020 flow diagram. Disagreements in full-text assessment will again be resolved by consensus or, if necessary, third-reviewer adjudication (Figure 2).

### Data extraction

A standardized, pre-piloted data extraction form will be developed and tested on three eligible studies before full application to the entire included dataset. The form will be constructed in Microsoft Excel. For each included study, the following data will be extracted: the names of authors and year of publication; the aim of the study and journal or source of publication; the country in which the study was conducted, the specific region or city, and the type of correctional facility involved; the study design; the dates of data collection and the overall study period; and the funding source. Population-level data to be extracted include the total sample size; the mean or median age of participants with the reported range or interquartile range; the sex and gender distribution; the type of inmates included (remand only, sentenced only, or both); the mean or median duration of incarceration with range.

The following outcome data will be systematically extracted from each eligible study: the overall prevalence of eye diseases expressed as a percentage with 95% confidence intervals; the prevalence of each specific ocular condition reported; visual acuity data including both presenting and best-corrected visual acuity; the classification of visual impairment and blindness according to either WHO criteria or the criteria used by the study authors; and all reported determinant or risk factor data including odds ratios, prevalence ratios, or relative risks with 95% confidence intervals and *p*-values.

### Data extraction process

Two reviewers (B.B.C and K.O.K) will independently extract data from each included study using the standardized form. Following independent extraction, the two data sets will be cross-checked item by item. Any discrepancies identified will be resolved through structured discussion between the two extracting reviewers. If agreement cannot be reached, the item in question will be referred to a third reviewer (JA) for final arbitration. Cohen’s kappa will be calculated and reported for the title/abstract screening. Where data are incomplete, ambiguous, presented in a non-extractable format, or not reported in the required form, the corresponding author(s) of the primary study will be contacted by standardized email with a specific data request, allowing a minimum two-week response window before the data are classified as unavailable.

### Quality assessment and risk of bias

The methodological quality of each included study will be appraised independently by two reviewers using the Joanna Briggs Institute (JBI) Critical Appraisal Checklist for Studies Reporting Prevalence Data. This tool evaluates key aspects of study quality including: appropriateness of the sampling frame, adequacy of the sampling method, sample size justification, subject description, completeness of data collection and analysis, sufficiency of the study sample, use of valid and reliable measurements for the outcome, appropriateness of statistical analysis, and response rate adequacy. Each domain will be scored as “yes”, “no”, “unclear”, or “not applicable”. An overall quality score will be derived as the proportion of criteria scored “yes” out of the applicable criteria; studies will be categorized as low (≤49%), moderate (50–69%), or high quality (≥70%) accordingly (27). Studies will not be excluded on the basis of quality assessment alone, but quality scores will be incorporated into sensitivity analyses. Domain-level JBI ratings will be presented for all included studies; aggregate scores will be used only for sensitivity analysis (to assess robustness when excluding low-quality studies) and will not serve as grounds for study exclusion. Additionally, traffic light plot will be used to visualized each domain and summary plot to ascertain the overall evidence. Further, the quality of included studies will be ascertained using the National Health Blood and Lung Institute (see link: https://www.nhlbi.nih.gov/health-topics/study-quality-assessment-tools) quality assessment tool. Agreement between reviewers during quality assessment will be evaluated using Cohen’s kappa statistic.

### Statistical analysis and data synthesis

#### Meta-analytic approach

Meta-analysis will be undertaken when at least three studies report sufficiently comparable outcomes and study characteristics. Given the anticipated substantial heterogeneity in study populations, prison environments, diagnostic approaches, and geographic settings, a random-effects meta-analysis using the DerSimonian-Laird method will be employed to compute pooled prevalence estimates. Prevalence data from each study will primarily be transformed using the Freeman-Tukey double arcsine transformation to stabilize variances and permit inclusion of studies reporting very low or very high prevalence estimates. However, if our prevalence values are not extreme and are symmetrically distributed, a logit transformation will serve as an alternative approach. For analyses involving a small number of studies (<5 studies per subgroup), the Hartung-Knapp-Sidik-Jonkman adjustment will be applied to reduce the risk of overly narrow confidence intervals. Studies reporting zero prevalence for a given outcome will not be excluded from the meta-analysis. The Freeman–Tukey transformation permits direct inclusion of such studies without continuity correction. Analyses will be conducted in R software (version 4.5.0) using the “meta” and “metafor” packages.

#### Assessment of heterogeneity

Statistical heterogeneity across studies will be assessed using the I^2^ statistic and Cochran’s Q test. I^2^ values will be interpreted as follows: 0–24% = negligible heterogeneity; 25–49% = low heterogeneity; 50–74% = moderate heterogeneity; ≥75% = considerable heterogeneity. A *p*-value of <0.10 for Cochran’s Q test will be considered indicative of significant heterogeneity. In the presence of considerable heterogeneity, subgroup analyses and meta-regression will be conducted to explore potential sources. If considerable heterogeneity (I^2^ ≥ 75%) persists after subgroup analyses and meta-regression and no clinically meaningful explanation can be identified, pooled prevalence estimates will not be reported for that outcome and findings will instead be summarized narratively. For all pooled prevalence estimates, 95% prediction intervals will be reported to indicate the expected range of prevalence estimates in future comparable prison populations. Potential overlap of prison cohorts will be assessed by examining study location, correctional facility names, recruitment periods, sample characteristics, and author affiliations. Where multiple reports arise from the same prison cohort, only the most comprehensive dataset or publication reporting the largest sample size will be retained.

#### Subgroup analyses

A minimum of three studies per subgroup will be required to proceed with any pre-specified subgroup analysis; where fewer than three studies are available, the subgroup will be described narratively only. Subgroup analyses will be conducted according to age group (adult versus juvenile detention populations), where sufficient data is available. In the event that the number of eligible studies is insufficient for all planned subgroup analyses, the following priority order is pre-specified: (1) diagnostic method (clinical vs. screening-only); (2) geographic sub-region; (3) sex composition; (4) prison type; and (5) study period. Findings from subgroup analyses will be interpreted cautiously and considered exploratory given the increased risk of spurious findings arising from multiple comparisons.

#### Sensitivity analysis

Sensitivity analyses will be conducted to examine the robustness of the pooled estimates by: (i) excluding studies with high risk of bias (low quality scores); (ii) excluding studies with larger sample sizes; and (iii) using leave-one-out analysis to assess the influence of individual studies on the overall pooled estimate. The effects of missing data will also be adequately assessed in sensitivity analysis. Additionally, the logit transformation will be applied as a pre-specified sensitivity analysis for all primary outcomes, regardless of the observed distribution of prevalence values. A continuity correction of 0.5 will be applied for zero-event cells under the logit sensitivity analysis.

#### Publication bias

Publication bias will be assessed if ten or more studies are available for any primary outcome, using funnel plot asymmetry (visually and statistically via Egger’s test for continuous outcomes and the Peters test for proportional data). If publication bias is detected, trim-and-fill analysis will be conducted to estimate the adjusted pooled prevalence.

#### Meta-regression

Where sufficient studies are available (*n* ≥ 10), univariable and multivariable meta-regression analyses will be performed to explore the association between prevalence estimates and potential moderating variables, including mean age of inmates, sex proportion, study year, country income level, and sample size.

#### Narrative synthesis

Irrespective of the feasibility of formal meta-analysis, a structured narrative synthesis will be conducted for all included studies and will constitute the primary vehicle for evidence integration. It will describe the distribution of study characteristics across countries, settings, and time periods; document patterns in the prevalence and type of ocular morbidity identified across the SSA region; summarize identified determinants and risk factors for ocular conditions; and characterize methodological themes, including the nature and sources of heterogeneity across the evidence base. Where quantitative synthesis is not appropriate, tabulated summaries of individual study findings will supplement the narrative. Where data are not permissible for statistical pooling or subgroup analyses, the Synthesis Without Meta-analysis (SWiM) guidelines will be applied to consolidate and present evidence thematically.

### Certainty of evidence (GRADE)

The overall certainty of the body of evidence for each primary and secondary outcome will be formally assessed using the GRADE (Grading of Recommendations Assessment, Development and Evaluation) approach. Under this framework, evidence will be classified as High, Moderate, Low, or Very Low certainty. Five domains will inform downgrading decisions: risk of bias in the contributing studies, as assessed through the JBI quality appraisal tools; inconsistency, reflecting the degree of unexplained heterogeneity in results across studies; indirectness, referring to the extent to which the populations, exposures, and outcomes studied in the primary evidence match those of the review question; imprecision, assessed on the basis of the width of confidence intervals and the adequacy of total sample sizes; and publication bias, as evaluated through funnel plot analyses. Upgrading of certainty may be considered where very large effects are consistently observed across high-quality studies or where a clear dose–response relationship is identified. GRADE evidence profiles and Summary of Findings (SoF) tables will be generated for all primary outcomes using GRADEpro GDT software (gradepro.org). The certainty ratings produced through this process will directly inform the strength and conditionality of the review’s conclusions and recommendations. Internal validity of our systematic review will be performed using AMSTAR-2 (A MeaSurement Tool to Assess Systematic Reviews - 2).

## Discussion

This protocol describes a systematic review and meta-analysis that will, for the first time, compile and quantitatively synthesize all available evidence on the prevalence of eye diseases among prison inmates in SSA. Individual country studies have collectively suggested that ocular morbidity is highly prevalent among this group; however, these estimates cannot be meaningfully compared or extrapolated without a synthesis that accounts for methodological differences, sampling approaches, and population characteristics. The proposed review will address this gap by generating pooled prevalence estimates and exploring sources of variability in a transparent and reproducible manner. By adhering to rigorous methodology and transparent reporting practices, the review will establish reliable estimates of the burden of eye disease across SSA, disaggregated by specific condition and geographic sub-region; characterize risk factors predisposing inmates to higher burden of eye diseases; critically appraise the methodological quality of existing studies; and highlight the unmet need for specialist eye care within SSA correctional facilities to support advocacy and policy reform.

The findings are expected to have direct implications for health policy and clinical practice across the region. Globally, the right to health of incarcerated persons is recognized under international law, including the International Covenant on Economic, Social and Cultural Rights and the UN Standard Minimum Rules for the Treatment of Prisoners (28). These instruments explicitly mandate that prisoners receive access to the same standard of healthcare available to the general population, without discrimination. In practice, however, access to specialist eye care within SSA prisons is profoundly limited or entirely absent in most facilities.

Should the review confirm that eye diseases are substantially prevalent among SSA prison inmates, the findings will constitute a compelling evidence base for policy reform. Specifically, the review may support recommendations for: the routine integration of eye health screening into prison intake health assessments; the establishment of basic ophthalmic services or periodic eye care outreach programs within prison facilities; provision of corrective lenses and reading glasses for inmates with refractive errors and presbyopia; referral pathways to secondary-level ophthalmology services for conditions requiring surgical or specialist management; nutritional supplementation strategies to mitigate diet-related ocular risks; and advocacy for reduced overcrowding as a structural determinant of communicable eye disease transmission. The review findings will also be relevant to national eye health program planners in SSA countries, many of whom are in the process of developing or revising national eye health action plans to align with the WHO World Report on Vision (29) and the IAPB 2030 global strategy (30). Prison populations are rarely explicitly included in national eye health planning, and the evidence generated by this review will provide a basis for their explicit inclusion as a priority group.

## Strengths and limitations

This systematic review has several methodological strengths that enhance its validity and utility. The review is the first of its kind to systematically address eye health among incarcerated populations across the entirety of SSA, filling a documented gap in the global eye health literature. The use of a comprehensive, multi-database search strategy maximizes sensitivity and reduces the risk of relevant studies being missed. The application of PRISMA reporting guidelines will ensure methodological transparency and reproducibility. The independent, dual-reviewer approach for screening, data extraction, and quality assessment introduces rigor and reduces the risk of selection bias and data extraction errors.

Some potential limitations of the review are acknowledged. First, the availability of published evidence on ocular health among incarcerated populations in SSA is expected to be limited, with substantial variation in the availability of country-level data. Many SSA countries may have little or no published evidence on prison eye health, which may limit the generalizability of pooled estimates and may result in overrepresentation of countries with stronger research capacity or better-established prison health systems.

Second, substantial heterogeneity across studies is anticipated due to differences in study design, sampling methodology, diagnostic approaches, definitions of eye conditions, and characteristics of incarcerated populations. In particular, variability in ophthalmic assessment methods may influence prevalence estimates and complicate comparisons across studies.

Additionally, ocular diseases may be underdiagnosed and underreported in correctional facilities with limited access to ophthalmic personnel, diagnostic equipment, or routine eye health screening services. As a result, studies conducted in settings with inadequate eye care infrastructure may underestimate the true burden of ocular morbidity among prisoners. Differences in healthcare access, facility resources, and urban–rural location of correctional facilities may further contribute to variation in reported prevalence.

The reliance on cross-sectional study designs limits causal inference, as temporal relationships between potential risk factors and ocular conditions cannot be established. Although an extensive search of peer-reviewed literature will be undertaken, grey literature sources will not be included. As a result, relevant unpublished studies, governmental reports, or institutional documents may not be captured, potentially introducing publication bias. However, restricting inclusion to peer-reviewed literature will enhance validity and rigor of the synthesized findings given its extensive methodological quality check from formal peer review. Finally, survivor bias may affect prevalence estimates if inmates with severe illness are transferred, released, or otherwise excluded from study populations.

## References

[1] Bourne R Steinmetz JD Flaxman S Briant PS Taylor HR Resnikoff S . Trends in prevalence of blindness and distance and near vision impairment over 30 years: an analysis for the global burden of disease study. Lancet Glob Health. (2021) 9:e130–43. doi: 10.1016/S2214-109X(20)30425-3, 33275950 PMC7820390

[2] Vision Loss Expert Group of the Global Burden of Disease Study and the GBD 2019 Blindness and Vision Impairment Collaborators. Prevalence of blindness and visual impairment in sub-Saharan Africa in 2020: magnitude and temporal trends. Systematic review and meta-analysis. Ophthalmic Epidemiol. (2026) 33:43–53. doi: 10.1080/09286586.2025.2474654 40127261

[3] Xulu-Kasaba ZN Kalinda C. Prevalence of the burden of diseases causing visual impairment and blindness in South Africa in the period 2010–2020: a systematic scoping review and meta-analysis. Trop Med Infect Dis. (2022) 7:34. doi: 10.3390/tropicalmed7020034, 35202229 PMC8877290

[4] Fentie D Solomon Y Menberu T. The burden of visual impairment among Ethiopian adult population: systematic review and meta-analysis. PLoS One. (2023) 18:e0288707. doi: 10.1371/journal.pone.0288707, 37471314 PMC10358928

[5] Asmare ZA Seifu BL Fente BM Negussie YM Asebe HA Bezie MM . Through the fog: systematic review and meta-analysis of the prevalence and associated factors of poor post-operative visual outcome of cataract surgery in sub-Saharan Africa. PLoS One. (2024) 19:e0315263. doi: 10.1371/journal.pone.0315263, 39652539 PMC11627423

[6] Favril L Rich JD Hard J Fazel S. Mental and physical health morbidity among people in prisons: an umbrella review. Lancet Public Health. (2024) 9:e250–60. doi: 10.1016/S2468-2667(24)00023-9, 38553144 PMC11652378

[7] Hewson T Minchin M Lee K Liu S Wong E Edge C . Interventions for the detection, monitoring, and management of chronic non-communicable diseases in the prison population: an international systematic review. BMC Public Health. (2024) 24:292. doi: 10.1186/s12889-024-17715-7, 38267909 PMC10809496

[8] Zalwango C Ayebare P Mwanja P Denis E Kasadhakawo M Mugerwa M . Prevalence and factors associated with ocular morbidity among prisoners of Luzira prison (Uganda). BMC Ophthalmol. (2021) 21:278. doi: 10.1186/s12886-021-02035-w, 34261442 PMC8278745

[9] Simooya OO Infections in Prison in Low and Middle Income Countries: Prevalence and Prevention Strategies. The Open Infectious Diseases Journal. (2010) 4, 33–37.

[10] Ayuk AA Chimaobi O Omang T Nwankwo EE. Continuous ex-offenders’ reformation and avoidance of recidivistic acts in Nigeria. Int J Criminol Sociol. (2020) 9:1631–7. doi: 10.6000/1929-4409.2020.09.187

[11] Mathenge W Kuper H Myatt M Foster A Gilbert C. Vitamin a deficiency in a Kenyan prison. Trop Med Int Health. (2007) 12:269–73. doi: 10.1111/j.1365-3156.2006.01780.x, 17300635

[12] Yilma Y Getachew M Mezemir Y. Assessment of under nutrition and its influencing factors among prisoners living with HIV/AIDS in north Shoa zone Amhara region Ethiopia, 2021. Mathews J HIV AIDS. (2021) 6:23.

[13] Topp SM Moonga CN Luo N Kaingu M Chileshe C Magwende G . Exploring the drivers of health and healthcare access in Zambian prisons: a health systems approach. Health Policy Plan. (2016) 31:1250–61. doi: 10.1093/heapol/czw059, 27220354 PMC5035781

[14] Ajite KO Adegbehingbe BO Omotoye OJ Ajayi IA Taiwo O. Prevalence of eye disease among inmates of Ilesa prison, Southwest Nigeria. Niger J Clin Med. (2011) 4. doi: 10.4314/njcm.v4i1.2

[15] Dixey R Nyambe S Foster S Woodall J Baybutt M. Health promoting prisons – an impossibility for women prisoners in Africa? Agenda. (2015) 29:95–102. doi: 10.1080/10130950.2015.1110943

[16] Okunlola PO Babatunde AO Akoki DM Ilori OT Femi-Lawal VO Abiona FM . Toward equitable health care: bridging the gap in the health of incarcerated individuals in Africa. Public Health Chall. (2024) 3:e70020. doi: 10.1002/puh2.70020, 40496426 PMC12039710

[17] Van Hout M-C Mhlanga-Gunda R. Prison health situation and health rights of young people incarcerated in sub-Saharan African prisons and detention centres: a scoping review of extant literature. BMC Int Health Hum Rights. (2019) 19:17. doi: 10.1186/s12914-019-0200-z, 31118008 PMC6532240

[18] Telisinghe L Charalambous S Topp SM Herce ME Hoffmann CJ Barron P . HIV and tuberculosis in prisons in sub-Saharan Africa. Lancet. (2016) 388:1215–27. doi: 10.1016/S0140-6736(16)30578-5, 27427448 PMC6182190

[19] Henostroza G Topp SM Hatwiinda S Maggard KR Phiri W Harris JB . The high burden of tuberculosis (TB) and human immunodeficiency virus (HIV) in a large Zambian prison: a public health alert. PLoS One. (2013) 8:e67338. doi: 10.1371/journal.pone.0067338, 23967048 PMC3743881

[20] Ekwenchi EE Ezegwui I Ezepue U Umeh R. Pattern of eye disorders among inmates of a Nigerian prison. Orient J Med. (2010) 21:1–4. doi: 10.4314/ojm.v21i1-4.54468

[21] Naidoo K Kempen JH Gichuhi S Braithwaite T Casson RJ Cicinelli MV . Prevalence and causes of vision loss in sub-Saharan Africa in 2015: magnitude, temporal trends and projections. Br J Ophthalmol. (2020) 104:1658–68. doi: 10.1136/bjophthalmol-2019-315217, 32229517

[22] Xulu-Kasaba ZN Kalinda C. Prevalence of blindness and its major causes in sub-Saharan Africa in 2020: a systematic review and meta-analysis. Br J Vis Impair. (2020) 40:563–77. doi: 10.1177/02646196211055924

[23] Mensah RO Boateng W Opoku D. Exploring the perceptions between ageing and mortality in selected prison facilities in Ghana. Discov Glob Soc. (2025) 3:14. doi: 10.1007/s44282-025-00156-x

[24] Moher D Shamseer L Clarke M Ghersi D Liberati A Petticrew M . Preferred reporting items for systematic review and meta-analysis protocols (PRISMA-P) 2015 statement. Syst Rev. (2015) 4:1. doi: 10.1186/2046-4053-4-1, 25554246 PMC4320440

[25] Parums DV. Review articles, systematic reviews, meta-analysis, and the updated preferred reporting items for systematic reviews and meta-analyses (PRISMA) 2020 guidelines. Med Sci Monit. (2020) 27:e934475-1. doi: 10.12659/MSM.934475 PMC8394590 34421116

[26] Babineau J. Product review: Covidence (systematic review software). J Can Health Libr Assoc. (2014) 35:68–71. doi: 10.5596/c14-016

[27] Saletta JM Garcia JJ Caramês JMM Schliephake H da Silva Marques DN. Quality assessment of systematic reviews on vertical bone regeneration. Int J Oral Maxillofac Surg. (2019) 48:364–72. doi: 10.1016/j.ijom.2018.07.014, 30139710

[28] Pinto M. "International covenant on economic, social and cultural rights". In: United Nations Audiovisual Library of International Law, vol. 11 (2022).

[29] World Health Organization. World report on vision. Geneva: WHO. (2019). Available online at: https://wkc.who.int/resources/publications/i/item/world-report-on-vision. (Accessed June 8, 2026).

[30] World Health Organization. Package of eye care interventions. (2022). Available online at: https://www.who.int/publications/i/item/9789240048959. (Accessed June 8, 2026).

